# They Built My Soul: A Qualitative Analysis of the Impacts of Home Repairs in Rural Tennessee

**DOI:** 10.13023/jah.0401.03

**Published:** 2022-02-13

**Authors:** Bethseda O’Connell, Ada Sloop, Nicole Intagliata, Megan Quinn

**Affiliations:** Department of Community and Behavioral Health, East Tennessee State University; East Tennessee State University; Appalachia Service Project; Department of Biostatistics and Epidemiology, East Tennessee State University

**Keywords:** Appalachia, housing, substandard, social determinants, rural

## Abstract

**Background:**

Housing is an important social determinant of health and substandard housing is linked to physical, mental, and social health problems.

**Purpose:**

The purpose of the study was to qualitatively assess the impacts of repairs to substandard housing in rural East Tennessee through twenty-eight interviews.

**Methods:**

Zoom was utilized for recording phone interviews in January–February 2021 and NVivo software was used for thematic analysis in May–July 2021.

**Results:**

Themes that emerged included environmental risk reduction, impacts on physical health, impacts on mental health, impacts on financial well-being, and willingness to receive future assistance from service organizations.

**Implications:**

Further research is recommended to quantify impacts including effects on utilization of health care and community services, school and work attendance, and mental health impacts.

## INTRODUCTION

Housing is among the leading social determinants of health.^1^ The Centers for Disease Control and Prevention defines social determinants of health as conditions in the places where people live, learn, work, and play.^2^ According to the US Department of Housing and Urban Development, a home is considered substandard if it is dilapidated, and/or lacks operable plumbing, a functional flush toilet, a functional bathtub or shower, safe electricity, a safe or adequate source of heat, a kitchen, or has been deemed unfit by an agency or unit of government.^3^ Individuals with substandard housing are potentially exposed to pest infestations, mold, leaks and dampness, poor ventilation, noise pollution, injury hazards, extreme temperatures, inaccessibility, poor sanitation and hygiene, lead, air pollutants and allergens.^4^,^5^ Therefore, substandard housing can result in increased risks including falls, unintended injuries, anxiety, social isolation, stress, allergy conditions such as asthma, respiratory diseases, cardiovascular diseases, and infectious diseases.^4^

Previous studies indicate that improvements to housing impact health, well-being, and poverty. A review indicated that increases in warmth and energy efficiency (such as insulation and fixing leaks) in “inadequate” housing results in improved overall health, respiratory health, mental health, social well-being, and attendance at school or work.^6^ A study examining “cold homes” (energy inefficient and hard-to-heat homes) suggested that energy efficiency improvements impact well-being and quality of life, financial stress, thermal comfort, social interactions, and indoor space use.^6^ Modifications to improve accessibility to and in homes are evidenced to increase safety and confidence, mobility, independence, social participation, ability to return home from hospitalization, and supports the role of caregivers.^7^ Additionally, a broad range of housing interventions were evidenced to produce “health gains.”^8^

Housing disparities are potentially more persistent and have greater health effects in areas that have higher poverty rates and poor health. The Appalachian region struggles economically and socially, resulting in health disparities.^9^ Central Appalachia has a poverty rate well above the national figure, with 18.2 percent of its households below the poverty line.^10^ In Appalachia, poverty rates increase as rurality increases.^10^ The four counties included in this study are composed of mostly rural populations, and classified as at-risk or distressed counties in northeast Tennessee.^10^ Lower economic status is reflected in poorer living conditions in the region than in the nation as a whole.^10^ Additionally, Appalachian states are some of the least healthy in the nation, with Tennessee ranking 44^th^ overall out of the 50 states in 2019.^9^

The Appalachian population has been characterized by a desire for self-sufficiency, a desire to preserve their autonomy, resistance to governmental authority, resistance toward assistance programming, and distrust of outsiders.^11^–^14^ Some of these cultural traits have historically compounded the struggle of programming to improve development in Appalachia, especially government-funded efforts.^12^,^13^ Private or nonprofit organizations may be more effective in housing efforts.^15^

The purpose of this study was to conduct participant interviews to examine the impacts of housing repairs conducted by Appalachia Service Project in Northeast Tennessee. This study is a pilot study and findings are intended to inform further research. Appalachia Service Project (ASP) is a nonprofit ministry with a vision to eradicate substandard housing in Central Appalachia.^16^ They provide critical repairs for over 350 families every year in Central Appalachia.^16^

## METHODS

Participants are selected for critical home repairs by ASP based on a variety of factors such as time availability, budget, type of repairs needed, volunteer skill level, amount of need in the surrounding county, location, proof of home ownership, and individual needs.^16^ Inclusion criteria for participation in this research project were having received housing repairs through ASP^16^ from 2017–2019, being at least 18 years old, and residence in Hancock, Cocke, Johnson, or Washington counties, Tennessee. Individual socioeconomic and demographic information were not collected as part of this research process in order to protect participants’ privacy. ASP provided the research team a list of phone numbers for 139 households eligible to participate. Participants were recruited to the study via phone call and verbally consented to participate in an interview. The study was exempt by the East Tennessee State University Internal Review Board, and the study adhered to ethical research practices.

Twenty-eight structured interviews (response rate=20.14%) were conducted about the participants’ experience with the housing repairs process. Questions included requests for description of the repairs and improvements, overall experience, physical, emotional, social, and financial impacts on the participants and their families, and willingness to request assistance. See Appendix 1 (in Additional Files) for the interview guide. The interviews were conducted using the Zoom phone call feature and were audio recorded with permission from the participants. Because no video is included in the Zoom call feature, access to internet was not required for participation. Transcription was completed using the Zoom transcription tool, and edits were made by interviewers using the recordings. There were two interviewers who transcribed their own interviews.

Thematic analysis of the interview transcripts was conducted by one researcher using NVivo software. Additionally, a word cloud was created using NVivo to provide a visualization of the most common words used by participants.

## RESULTS

Participants reported repairs or building of stairs, ramps, bathrooms, insulation, roofs, ceilings, floors, walls, porches, and railings. Themes were categorized into the following six groups: environmental risk reduction (n=24, 85.7%), impacts on physical health (n=19, 67.8%), impacts on mental health (n=26, 92.8%), impacts on financial well-being (n=15, 53.6%), and willingness to receive assistance from service organizations (n=20, 71.4%). Participants could mention more than one sub-theme within overarching themes. Summaries of each theme with sub-themes and illustrative quotes are provided in the sections below.

### Environmental Risk

Reported environmental risk reduction included reduced fall risk (n=8), less leaks and moisture (n=6), improved climate control (n=5), less mold (n=4), improved sanitation and cleanliness (n= 4), and fewer pests (n=1). Many of these impacts are directly related to the “warmer, safer, drier” mantra of the intervening organization.^16^ Example quotes about risk reduction are displayed in Table 1.

### Physical Health

Specific health problems suffered by participants and family members included allergies, COPD, “smoker’s cough,” lupus, arthritis, cancer, injuries, an amputation, and even death. Impacts on physical health were noted by 19 participants. Some participants attributed health improvements directly to repairs (n=7). The most common physical health impact was increased accessibility (n=12), making coping with health problems easier. Increased accessibility was noted both within the home and while entering/exiting the home. Table 2 includes quotes relevant to impacts on physical health.

### Mental Health

The most frequently reported impacts were on mental health, with 79 references to various mental health impacts by nearly all participants (n=26). Mental health impacts included appreciation that someone cared (n=8), reduced anxiety or worry (n=7), enjoying improved aesthetics (n=7), general emotional uplifting (n=6), no longer being embarrassed to have guests visit (n=4), hope for the future (n=3), and spiritual encouragement (n=1). Additionally, there were 39 references to participants enjoying their interaction with ASP staff and volunteers, citing being treated with kindness (n=19), respect (n=11), positive social interactions (n=12), and positive and professional communication (n=4). The positive experience and respect were linked to willingness to receive assistance (described in more detail within the Willingness to Receive Assistance theme). Table 3 includes quotes about impacts of home repairs on mental health.

### Willingness to Receive Assistance

Most participants (n=20) stated that they would be willing to receive assistance from a service organization in the future. Many voiced hesitance or not having previously received assistance other than through ASP. Participants tied willingness to receive assistance to the positive experience with ASP and being treated with respect. See Table 4 for quotes about willingness to receive assistance.

### Financial Well-being

Impacts on financial well-being were reported by participants, including that they would have otherwise been unable to afford the work (n=13), reduced cost of utilities (n=7), prevented debt (n=2), and increased property value (n=1). This theme seemed to be linked to impacts on mental health as the financial benefits were a relief to clients.

When asked what could have been better about their experience, most participants had no criticisms. There were few suggestions for improvement (n=9), mostly that participants had hoped for more work to be done, something they wanted repaired could not be done, or a problem was not completely solved.

**Figure 1:**
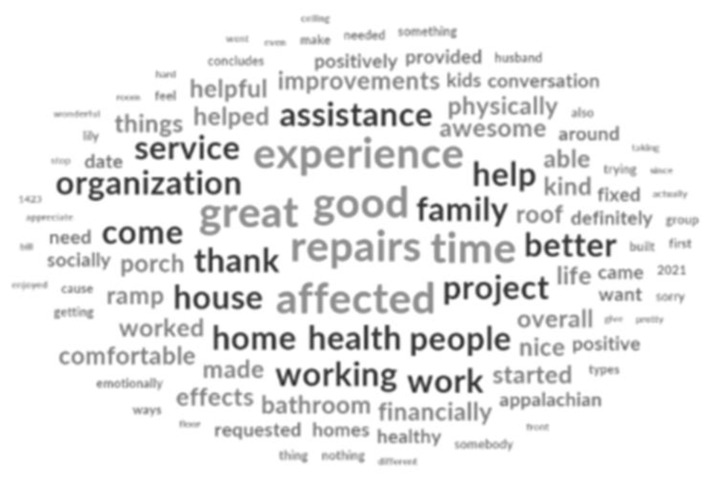
Transcript Word Cloud

A word cloud was created to visually represent the content of the interview conversations. Word clouds are graphical representations of the frequency of word use, with larger words being used more frequently. Some of the most common words used by participants included good, great, better, experience, time, work, repairs, project, assistance, help, affected, health, healthy, house, people, family, and thank. See Figure 1 for the word cloud.

## IMPLICATIONS

This study found that the impacts of repairs to substandard housing in rural Northeast Tennessee are far-reaching to include themes related to environmental, physical, mental, and financial health as well as willingness to receive assistance. These findings have implications for medical care, mental health care, economics, public health, and social work, and other related professions. Interventions in these fields must also consider home environments. One way to do this may be consistent use of social determinants screening including housing and a method of referral to appropriate services. These findings support the need for repairs to substandard housing and the need for policies and funding to support such interventions. Additional research is needed to further understand the specific impacts on the study population and to quantify those impacts. Further research could investigate measures of these broad impacts such as utilization of health care and community services, school and work attendance, and mental health impacts including reduced anxiety and social isolation.

Study strengths include the broad qualitative nature of the questions in order to describe the breadth of impacts of the interventions and providing the opportunity for the population’s perspective to be heard. Study limitations include potential recall and self-report biases. Potential social desirability bias was limited by asking broad and general questions in order to elicit responses that were not suggested or influenced by the interviewers and by utilizing university interviewers external to ASP. Additionally, the study population was limited to a small geographic region and small sample size and cannot be generalized.

**SUMMARY BOX**

**What is already known about this topic?** Housing is known to be an important social determinant of health, and Appalachia is known to disproportionately suffer economic and social burdens leading to health disparities.

**What is added by this report?** This study provides a broad overview of impacts of substandard housing in northeast Tennessee from the population’s perspective, including environmental risk reduction, physical health, mental health, financial well-being, and willingness to receive assistance from service organizations.

**What are the implications for future research?** These impacts require further research to fully understand the consequences to health and health systems.

## Supplementary Information



## Figures and Tables

**Table 1:** Quotes about environmental risk reduction

“We now have home safety”
“It was fantastic to be able to walk on the floor…and not be afraid of falling or slipping”
“They built steps for us because we had… actually cider blocks with palettes…very unsafe”
“no more rain in the bathroom”
“Well, I’m not standing at my stove with my teeth chattering trying to cook”
“It’s warmer! You don’t know how much you miss a warm house until you don’t have”
“I was really concerned about the black mold in the back bedroom”
“With being able to take a shower more often, it helps”

**Table 2:** Quotes about impacts on physical health

“We’re not getting sick as often because it’s not cold in the house.”
“We have been less prone to get sick, just because of the lack of the leaky roof, the new sheet rock on the walls, …the insulation in the ceiling.”
“There’s no black mold and we’re breathing better…”
“I was very limited of going and coming, you know, and after this I’ve got a buggy and I put my groceries in it and push it up the ramp… it turned my life around.”
“They put a rail in so it’s easier to step in and out of the tub… because I wasn’t able to get in and out of the tub because of my physical issues.”

**Table 3:** Quotes about impacts on mental health

“They don’t just build you a ramp, they built my soul.”
“Family and friends come in and visit and we’re not embarrassed or anything about how our house looks… so socialize more.”
“[The house] was stressing us out and we don’t worry about that now…it lifted a great burden.”
“gave me…hope in humanity again”
“Everyone was real nice and helpful and just respectful of our home.”

**Table 4:** Quotes about willingness to receive assistance

“No, I ain’t never asked for nothing [before]…yeah, I would, you know, [in the future] because I know it’s real and there ain’t nobody trying to steal or do nothing bad to you.”
“Yes ma’am, because of the positive experience we had with ASP. Yes ma’am, I would be more apt to ask, or you know, inquire about help elsewhere.”
“They don’t make you feel like you’re a charity case.”
“They didn’t make us feel like we were poor, couldn’t do anything ourselves.”
