# Supplementary material for: They Built My Soul: A Qualitative Analysis of the Impacts of Home Repairs in Rural Tennessee
Source: J Appalach Health. 2022 Feb 13;4(1):9–19. doi: 10.13023/jah.0401.03 (PMC9200454; doi:10.13023/jah.0401.03)
Supplement: Supplementary file 1 [file 4.1.3B_OConnell_Additional_File_1.pdf]

Appendix: Interview Guide

Introduction

Hello! My name is _____ and I am calling to talk with you about your experience with Appalachia Service Project (ASP). East Tennessee State University has partnered with Appalachia Service Project to help them learn more about your experience with home repairs through this program. You were selected for this interview because you have been an ASP client and living in _____ county.

I will be asking questions about your experience that we hope you will respond to. There are no wrong answers. Keep in mind that we're just as interested in negative comments as positive comments. We want you to feel comfortable participating. We will not associate your name with anything you say during the interview.

It is difficult for me to take notes on everything that is said here. So, we will also be recording the session so that we may transcribe it later. If you have any questions later, you can call Dr. Beth O'Connell at xxx-xxx-xxxx.

Participation is voluntary. You may refuse to answer any question or stop the interview at any time.

This interview is for research purposes. This information will only be shared with ETSU study staff. We will do everything we can to respect your privacy and protect your confidentiality.

The interview should take about 30-45 minutes depending on your answers and you answer only the questions you want to. By continuing with the interview, you will be consenting to take part in the research. May I ask you a few questions?

- [If no] okay, is there a better time that I can call back?
- [If yes] Would you lack to answer now, or schedule a time that is more convenient?

    Schedule call back time or

    Proceed with interview

- [If no] Ok, we will remove you from our interview list. Have a good day!

Are there are any questions before getting started?

Questions

1. What types of home repairs or improvements were done at your home through ASP?
2. Tell me about your experience with the Appalachian Service Project.
3. What about this experience was good?
4. What about this experience could have been better?
5. How have these repairs positively affected your life? Have they had any negative affects?
6. Probe: Have the repairs affected you or your family physically?
    a. Probe: Have the repairs affected you or your family emotionally?
    b. Probe: Have the repairs affected you or your family socially?
    c. Probe: Have the repairs affected you or your family financially?
7. Tell me about your overall health before working with ASP?
   After?

8. Before working with ASP, had you ever requested assistance from a service organization before? What was that experience like?
9. Now that you have worked with ASP, would you be more comfortable asking an organization for assistance?
10. Is there anything else you want to add to this conversation?

Conclusion

Thank you so much for your time. The information you provided will be very helpful to this project and to ASP. This concludes the interview.
